# Don’t wait for Paris summit to improve health 

**DOI:** 10.2471/BLT.15.031115

**Published:** 2015-11-01

## Abstract

How developing countries will make the transition to sustainable clean-energy economies is a major challenge for the United Nations summit that opens in Paris this month. Christiana Figueres talks to Andréia Azevedo Soares.

*Q: How did you become interested in climate change policy? *

A: As a child, my parents took me to a beautiful rainforest where there were these fantastic little golden toads. The species was endemic to the Monteverde Cloud Forest of Costa Rica. But by the time I had my own children the species had disappeared. I was deeply affected by this and did not want to leave to my children a greatly diminished planet compared with the one I inherited from my parents. Today there is some debate about the cause of the demise of the toads *Bufo periglenes*, but at the time this was the catalyst for the journey I have taken ever since. 

Q: You studied anthropology, which is a discipline that deals with human behaviour. How is this helping you to bring 195 nations together to deliver a new agreement? 

A: My anthropological background has been very helpful. I tend to see the world through that kind of a lens and that helps me appreciate and respect the many differences between people around the world. Each culture is so unique and embedded in its own history, natural resources and challenges. At the same time, we all live together on the same planet, so the anthropological approach underscores the changes we must make so that we can co-exist and collaborate. So I learned both the appreciation of differences and the pursuit of cooperation in the spirit of change from anthropology. 

Q: The debate about global warming has largely been an economic one. What progress has been made to get health higher up on the agenda? 

A: Dr Margaret Chan, the Director-General of World Health Organization (WHO), was one of the first global leaders to state clearly the linkages between climate change and health. Now the world is waking up to her warnings and progress has been made. First, there is much greater awareness of how burning fossil fuels, particularly coal, harms human health. Around 7 million people died in 2012 because of air pollution, according to WHO. People in Beijing and other cities suffer the painful consequences of very poor air quality. The good news is that China has started to reduce coal burning and is committed to a substantial reduction by 2020. Two decades ago this would have been unimaginable. Second, changing weather patterns are altering the distribution of insect populations and affecting the transmission of tropical diseases, in particular water- and vector-borne diseases, thus tropical diseases such as malaria and dengue are advancing in many parts of the world. Third, the advance of climate change – if we aren’t able to address it – will continue to affect food and water security globally, particularly in tropical zones, which is a direct threat to health. 

Q: Recent news about the car industry shows that combustion engines in industrialized countries may not be reducing carbon emissions as much as they should do. What can countries do to make anti-pollution regulations more effective? 

A: You need a balance between carrots and sticks. In the case of Volkswagen perhaps the carrots were too plentiful. In the end regulators identified the gulf between ‘tested’ and real emissions and the company is now having to deal with the consequences.

Q: Will we hear more about health at the climate summit in Paris than we heard at previous talks in Copenhagen in 2009, when health was mentioned once in the 200-page draft? 

A: We have been hearing about health and climate change for several years now and I am very grateful for that because the links between the two have become clear. Health ministers realize it’s in their interests to help their colleagues – the energy, finance and environment ministers – to address climate change because of the health issues. The 2015 *Lancet *Commission on Health and Climate Change, for example, was a real clarion call to the world’s ministries of finance and energy, not only ministries of health. They need to understand how interlinked their sectors are to introduce policies and measures that will address climate change and improve health conditions at the same time. A new, universal climate change agreement will bring countless co-benefits for health, but we don’t need to wait for Paris: acting on climate change and shifting to sustainable development has very clear benefits for public health. 

Q: What will be the greatest challenges in the forthcoming summit in Paris? What needs to happen for the agreement to be reached?

A: We will have an agreement. Every single country is focused on that. However, not everything is settled yet. One of the greatest challenges is: how to support developing countries in the transformation they need to undertake, given their growing populations and demands for energy. Developing countries are the first and hardest hit by the adverse effects of climate change. They need to figure out how to continue lifting their people out of poverty and achieve the living conditions that others in high-income countries enjoy, without burning fossil fuels but rather by integrating renewable energy into their grids. We are asking a lot because developed countries did this by burning fossil fuels. Now the developed countries are telling developing countries to continue developing, to improve their public transport and energy systems without fossil fuels. Developing countries need technical and financial support to make that transition. Adaptation measures in developing countries can be either infrastructural (constructing sea-level rise defences, or boosting the health and coverage of mangrove forests for instance) or based on behavioural shifts (encouraging people to use less water or follow contingency plans). Ensuring greater access to education and health facilities in low-income settings can also be a way of anticipating the adverse effects of climate change and trying to minimize the impact of these. 

Q: You mentioned that low-income countries are hardest hit by the ill-effects of climate change largely caused by industrialized countries. Are the governments of industrialized nations prepared to compensate the developing world? 

A: [Governments of] industrialized countries are prepared to assist for many reasons, but perhaps the most fundamental is the notion that none of us lives in isolation. We do not want our planet to be devastated; no one wants increasingly frequent and more intense extreme weather events to disrupt food supply chains or damage telecommunications networks. So policy-makers in industrialized countries are looking at how they can support developing countries. This comes at a time of fiscal austerity, a difficult time for many. But these policy-makers also know that short-term issues should not trump long-term objectives in the climate change negotiations.

“Developing countries are the first and hardest hit by the adverse effects of climate change.”

Q: You have said in the past that the involvement of women is key to fighting climate change, particularly in the developing world. Why? 

A: Women have the potential to drive behavioural change. Traditionally, we, women, determine family customs and culture that are passed on from generation to generation. In developing countries, we are often responsible for food production. Women often determine what food is put on the table – whether we produce it ourselves or whether we are the purchasers. Water, food and energy are also mainly in female hands in developing countries. So we are very much at the heart of the climate change discussion. 

Q: You founded the non-profit Centre for Sustainable Development of the Americas 20 years ago. What was it like campaigning in those days? 

A: It was a fantastic nongovernmental organization (NGO) devoted to working with governments and the private sector in Latin America to raise awareness of climate change and of the opportunities that solutions to climate change can bring. It was in the 1990s, when few people had even heard of climate change and I was often regarded with disdain. But, by the time we closed the NGO, we were quite pleased with the results and the fact that Latin America had started to take the lead in the United Nations climate change convention.

“Keeping to below the two-degree Celsius threshold before the end of the century is possible.”

Q: What is the significance of the Pope’s encyclical on climate change and what affect could this have on governments?

A: The importance of Pope Francis’s environmentally conscious statement of doctrine should not be underestimated. The encyclical is special because it brings together three imperatives: the scientific, the moral and the economic imperatives to take action on climate change. The Pope called for a “radical change” in our relationship with the Earth and its creatures, reflecting humanity’s God-given responsibility as custodians of the Earth. The encyclical is important because the Pope’s position as leader of 1.2 billion Catholics gives him the unique ability to unite campaigners working towards a common goal. The Pope is not the only religious leader to link poverty and climate change. Others include Islamic scholars, and leaders in the Orthodox church and Protestant faiths.

Q: Is the aim of holding global warming to less than two degrees Celsius over the pre-industrial average realistic? 

A: Keeping to below the two-degree Celsius threshold before the end of the century is possible and, if there is real, sustained and rising ambition in coming years, an even lower level could be achieved. Climate science shows us that to achieve this, the world needs to embrace a three-part plan: first, see global emissions peak in the next decade, second, drive them down so low to recapture the ecological balance between emissions and the natural absorptive capacity of the planet, a state known as climate neutrality and, third, become fully climate neutral within the second half of this century. A successful response to climate change will span several generations. Our task in Paris is to reach a credible, measurable and actionable plan that has an impact now and over time, one that is clear on how to provide the financial assistance for the poorest and most vulnerable to build their own clean and sustainable futures.

